# Phosphorylation Controls the Localization and Activation of the Lumenal Carbonic Anhydrase in *Chlamydomonas reinhardtii*

**DOI:** 10.1371/journal.pone.0049063

**Published:** 2012-11-06

**Authors:** Amaya Blanco-Rivero, Tatiana Shutova, María José Román, Arsenio Villarejo, Flor Martinez

**Affiliations:** 1 Department of Biology, Universidad Autónoma de Madrid, Madrid, Spain; 2 Umeå Plant Science Centre, Department of Plant Physiology, Umeå University, Umeå, Sweden; University of Hyderabad, India

## Abstract

**Background:**

Cah3 is the only carbonic anhydrase (CA) isoform located in the thylakoid lumen of *Chlamydomonas reinhardtii*. Previous studies demonstrated its association with the donor side of the photosystem II (PSII) where it is required for the optimal function of the water oxidizing complex. However this enzyme has also been frequently proposed to perform a critical function in inorganic carbon acquisition and CO_2_ fixation and all mutants lacking Cah3 exhibit very poor growth after transfer to low CO_2_ conditions.

**Results/Conclusions:**

In the present work we demonstrate that after transfer to low CO_2_, Cah3 is phosphorylated and that phosphorylation is correlated to changes in its localization and its increase in activity. When *C. reinhardtii* wild-type cells were acclimated to limiting CO_2_ conditions, the Cah3 activity increased about 5–6 fold. Under these conditions, there were no detectable changes in the level of the Cah3 polypeptide. The increase in activity was specifically inhibited in the presence of Staurosporine, a protein kinase inhibitor, suggesting that the Cah3 protein was post-translationally regulated via phosphorylation. Immunoprecipitation and *in vitro* dephosphorylation experiments confirm this hypothesis. *In vivo* phosphorylation analysis of thylakoid polypeptides indicates that there was a 3-fold increase in the phosphorylation signal of the Cah3 polypeptide within the first two hours after transfer to low CO_2_ conditions. The increase in the phosphorylation signal was correlated with changes in the intracellular localization of the Cah3 protein. Under high CO_2_ conditions, the Cah3 protein was only associated with the donor side of PSII in the stroma thylakoids. In contrast, in cells grown at limiting CO_2_ the protein was partly concentrated in the thylakoids crossing the pyrenoid, which did not contain PSII and were surrounded by Rubisco molecules.

**Significance:**

This is the first report of a CA being post-translationally regulated and describing phosphorylation events in the thylakoid lumen.

## Introduction

Carbonic anhydrases (CAs) serve different functions in the metabolism of algae and plants. Their role in carboxylation/decarboxylation reactions made them essential in algal carbon-concentrating mechanisms, ion transport, pH homeostasis and in the production of carbon skeletons by mitochondria (for reviews, see [1], [2], [3]). In *C. reinhardtii*, there are at least 12 genes that encode CA isoforms, including three alpha, six beta, and three gamma or gamma-like CAs [1].

Cah3, identified in *C. reinhardtii*, was the first intracellular alpha-CA described in algae and found to be localized in the thylakoid lumen [4],[5]. According to the thorough revision of carbonic anhydrase isoforms of *C*. *reinhardtii* recently made by Moroney *et al.* [1], this Cah3 is the unique isoform located on the lumenal side of thylakoid membranes. Two different models to explain its function have been proposed. Villarejo *et al.* [6] clearly stated that the Cah3 protein was functionally associated with the electron donor side of photosystem II (PSII), which is the site of proton release and O_2_ production. Intact cells, thylakoids and PSII-enriched membrane fragments of a mutant lacking Cah3 (*cia3*) showed impaired water-splitting ability [6]. Shutova *et al.* [7] further proposed that Cah3 enhances O_2_ evolution by locally providing HCO_3_^−^ as proton carrier to remove protons from the Mn complex.

However, it has also been suggested that Cah3 is functioning in the CO_2_-concentrating mechanism (CCM). In fact, all the mutants lacking Cah3 grow photo-autotrophically on high-CO_2_ levels but exhibit extremely poor growth under low-CO_2_ conditions [1]. All of them over-accumulate inorganic carbon, but they fail to assimilate it [8], [9]. At least one of those mutants, *cia3*, can be complemented by the wild type Cah3 gene [5].

According to Raven [10], the un-catalyzed conversion of bicarbonate to CO_2_, even at the low pH of the lumen, is at least 300 times too slow to support the observed high rates of photosynthesis. He postulated that the lumenal CA could be involved in accelerating the conversion of HCO_3_^−^ to CO_2_ in the lumen and thus stimulating the carboxylase activity of Rubisco [11]. In an attempt to explain the role of Cah3 and to distinguish between the two proposed models i.e. PSII function or CO_2_ supply for Rubisco, Hanson *et al.* [12] concluded that CO_2_ supply for Rubisco was the primary role of Cah3. Their results demonstrated that, after a short-term exposure to low CO_2_ conditions in the *cia3* mutant, CO_2_ fixation was already compromised whereas the same short exposure to low CO_2_ did not directly affect PSII activity [12].

On the other hand, the essential role of phosphorylation of proteins within the photosynthetic membranes for balancing the light distribution between PSII and PSI in plants and algae is now well established [13], [14], [15], [16], [17]. Two thylakoid-associated kinases, Stt7 and Stl1, have distinct roles in short and long-term photosynthetic acclimation to changes in light quality and quantity (state transitions), with Stl1 being in turn a phosphoprotein whose *in vivo* phosphorylation depends on Stt7 [16]. However, Stt7-independent protein phosphorylations have also been identified under different conditions [16].

Turkina *et al.* [18] reported the phosphorylation of two thylakoid proteins which occurred strictly at limiting CO_2_; their phosphorylation required reduction of electron carriers in the thylakoid membrane, but was not induced by light and both proteins were phosphorylated in the low-CO_2_-exposed stt7 deficient mutant. The latter was suggested as an early adaptive and signaling response of *Chlamydomonas* to the limited environmental inorganic carbon.

In this study we have characterized the biochemical processes involved in the CO_2_-responsive regulation of Cah3. We show here that this lumenal CA is phosphorylated during acclimation of *C. reinhardtii* cells to low CO_2_ conditions. We also provide strong evidence that phosphorylation of Cah3 causes activation of the enzyme and its redistribution to the PSII-devoid thylakoid membranes in the pyrenoid. This is the first report of a CA being post-translationally regulated via phosphorylation and describing phosphorylation events in the thylakoid lumen. We conclude that this mechanism allows algal cells to regulate the photosynthetic reactions by controlling the availability of HCO^3−^, not only at the early steps taking place in the water oxidizing complex [6], [7] but also at the end of the electron transport chain.

## Results

### The external CO_2_ concentration regulates the activity of the lumenal carbonic anhydrase Cah3

**Figure 1:**
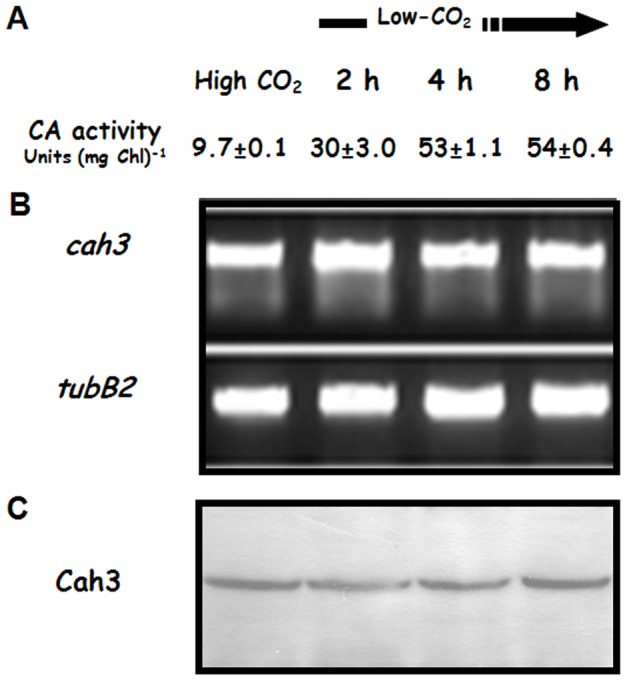
Analysis of thylakoid carbonic anhydrase (Cah3) activity and expression during the acclimation of high-CO_2_-grown *C. reinhardtii* cells to low CO_2_ conditions. (**A**) CA activity (WA units (mg Chl)^−1^) was measured in thylakoid membranes isolated from high-CO_2_-grown cells or acclimated to low CO_2_ for 2, 4 and 8 h. Values are means ± SE (*n* = 5). (**B**) Semiquantitative RT-PCR analysis of Cah3 gene expression. Total RNA to be used for RT was isolated by using Trizol™ reagent according to the manufacturers protocol (Life Technologies, US). Aliquots of the reaction mix were loaded and ethidium bromide stained in 1% agarose gels. (**C**) Immunoblot analysis of total cell extracts from cells of *C. reinhardtii* with antibodies raised against over-expressed Cah3 polypeptide. The lanes were loaded with 10 µg protein.

To obtain detailed information about how the CO_2_ concentration is regulating the lumen located Cah3 during growth of *C. reinhardtii* cells, we followed the changes in both activity of Cah3 and expression of the *cah3* gene after transferring cells from 5% (high CO_2_) to atmospheric CO_2_ levels (low-CO_2_; i.e. 0.035%). When high-CO_2_-grown cells of *C. reinhardtii* were transferred to low CO_2_ conditions, the lumenal CA activity increased (Figure 1A). The CA activity in thylakoid membranes from high-CO_2_-grown cells was 9.7 WA units/mg Chl (Figure 1A), while a 5- to 6-fold higher activity was observed with thylakoid membranes from low-CO_2_-grown cells. A similar increase in thylakoid CA activity has been previously reported in *Chlamydomonas* by using photoaffinity labelling and mass-spectrometric techniques [19], [20]. Our data clearly show that the activity increased to its maximum value within the first 4 h of acclimation to low CO_2_ conditions (Figure 1A), while longer exposure of the cells to low CO_2_ caused no further increase in the thylakoid CA activity (Figure 1A).

In order to analyze if the increase in CA activity was due to changes in gene expression, the levels of the Cah3 transcript were investigated by semi-quantitative RT-PCR (Figure 1B). These experiments revealed that the amount of the Cah3 transcript did not significantly change during the acclimation to low CO_2_ conditions (Figure 1B). In the same set of experiments, changes in the amount of Cah3 protein were not observed either (Figure 1C). Our results therefore suggest that the activation of Cah3 during the acclimation to low CO_2_ conditions may be caused by a post-translational regulation.

### Cah3 is phosphorylated during the acclimation to low CO_2_ conditions

**Figure 2:**
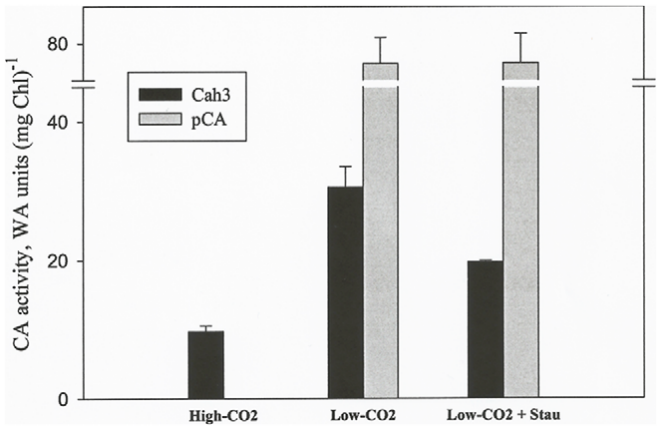
Staurosporine, a protein kinase inhibitor, partially inhibits the activation of Cah3 activity. High-CO_2_-grown *C. reinhardtii* cells were acclimated to low CO_2_ conditions for 2 h in the absence or in the presence of 0.1 µM Staurosporine. CA activity was measured in thylakoid membranes isolated from control and treated cells. As a control, periplasmic CA activity was measured using intact cells of the same cultures. Values are means ± SE (*n* = 5).

Phosphorylation/de-phosphorylation modifications of PSII polypeptides in the thylakoid membranes are now well established [13], [14], [15], [16], [17]. These modifications appear to be responsible for modulating the balance between PSII and PSI [21], [14]. To test if a protein kinase is involved in the activation of Cah3 during the acclimation to low CO_2_, *C. reinhardtii* cells growing under high-CO_2_ conditions were transferred to low CO_2_ in the presence of Staurosporine, a well-known inhibitor of eukaryotic Ser/Thr protein kinases [22]. In cells acclimated for 2 h to low CO_2_ in the presence of 0.1 µM Staurosporine the activation of the lumenal CA was inhibited by ∼40% when compared to that of control cells (19.7±0.3 and 30.5±0.8 WA units/mg Chl, respectively) (Figure 2). Neither the induction of a well-known low-CO_2_-inducible CA isoenzyme, like the periplasmic Cah1 (Figure 2), only expressed and active upon acclimation to low-CO_2_ [1], nor the expression of other low-CO_2_-inducible polypeptides (*data not shown*) was affected by the addition of the inhibitor. These results indicate that the activation of the lumenal located CA is, at least partly, under the control of phosphorylation/dephosphorylation mechanisms.

**Figure 3:**
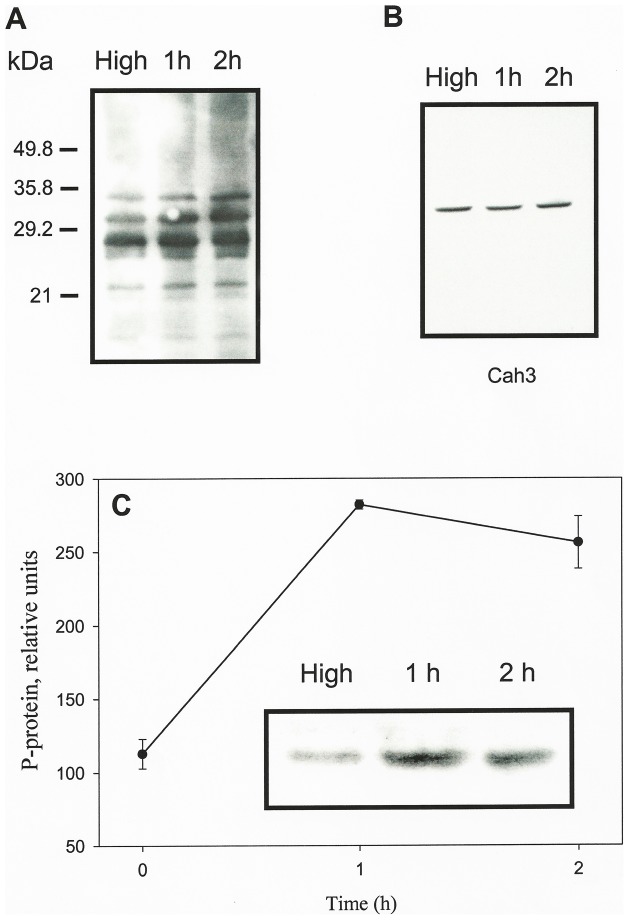
Phosphorylation of LHCIIP and PSII polypeptides during acclimation to low CO_2_ conditions. (**A**) Immunoblot analysis of thylakoid membrane proteins isolated from high-CO_2_-grown cells (H), and cells acclimated to low CO_2_ for 1 (1 h) and 2 h (2 h) probed with antibodies against phosphothreonine (Thr(P)). (**B**) Immunoblot analysis of extrinsic thylakoid proteins isolated from thylakoids of high-CO_2_-grown cells (H), and cells acclimated to low CO_2_ for 1 (1 h) and 2 h (2 h), probed with affinity-purified antibodies against Cah3. (**C**) Changes in the immunoresponse of Thr(P) antibody to a 30-kDa phosphoprotein during the acclimation to low CO_2_. The *inset* shows immunoblot analysis of extrinsic thylakoid proteins isolated from thylakoids of high-CO_2_-grown cells (H), and cells acclimated to low CO_2_ for 1 (1 h) and 2 h (2 h), probed with Thr(P) antibodies. The lanes were loaded with 10 µg protein.

In a first approach we analyzed the changes in the phosphorylation pattern of thylakoid membranes upon acclimation to low CO_2_ conditions by using phosphothreonine (Thr(P)) antibodies. This immunological approach has been introduced in the late 90s as an alternative tool to study *in vivo* phosphorylation processes which allows overcoming the limitations to detect changes in the level of endogenous phosphorylation of other currently used methods [23]. Our analysis revealed a rather complex pattern of major phosphoproteins which fit with the many thylakoid phosphopeptides that have been previously reported in *C. reinhardtii* [18], [24], [16], (Figure 3A). Therefore, to avoid the Cah3 protein being hidden by other major thylakoid phosphoproteins we carried out a similar analysis on fractions containing only extrinsic thylakoid proteins, based on the fact that, in *Chlamydomonas*, Cah3 is peripherally associated with the lumen side of the thylakoid membranes and can be released from the thylakoids using treatments that will not cause the release of the integral thylakoid phosphoproteins [5].

Extrinsic fractions were obtained from thylakoid membranes of *Chlamydomonas* cells either grown under high-CO_2_ conditions or acclimated to low CO_2_ for 1 and 2 h. Immunoblot analysis shows that Cah3 was recovered in all of the supernatants (Figure 3B). One major phosphoprotein at 30 kDa was present in these samples (Figure 3C, inset). A sharp increase (up to 3-fold) in the immunological cross-reactivity of this protein with the Thr(P) antibodies was taking place within the first hour of acclimation to low CO_2_ conditions (Figure 3C). Longer exposure of the cells to low CO_2_ caused no further increase of the phosphosignal.

**Figure 4:**
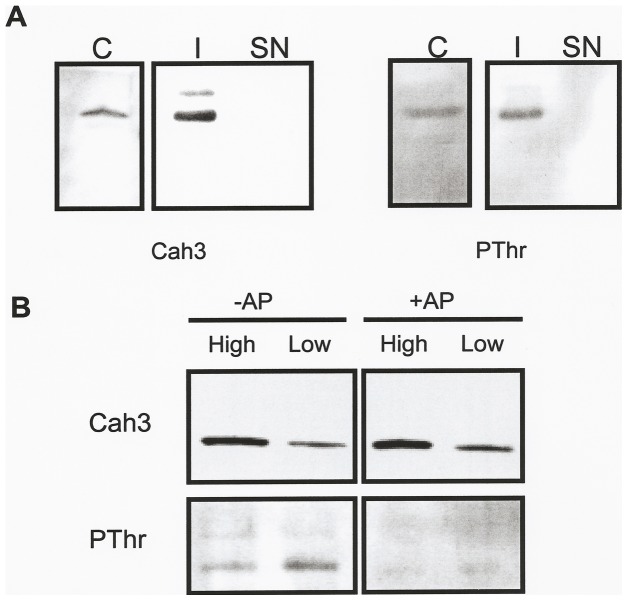
Immunoprecipitation and dephosphorylation experiments of extrinsic thylakoid polypeptides. Extraction of extrinsic thylakoid proteins was accomplished by washing the thylakoid membranes with a medium containing low concentrations (0.05%) of Triton X-100. (**A**) The 30-kDa extrinsic phosphoprotein immunoprecipitates with Cah3. Extrinsic thylakoid proteins released from thylakoid membranes of *C. reinhardtii* cells acclimated to low CO_2_ for 2 h (C) were immunoprecipitated with affinity-purified antibodies against Cah3 and protein A-Sepharose CL-4-B beads. The Sepharose beads were washed and the immunoprecipitate (I) and the supernatant (SN) obtained after centrifugation were analysed by SDS-PAGE and immunoblot and probed with antibodies against Cah3 (left) and Thr(P) (right). (**B**) Effect of Alkaline phosphatase (AP) treatment on extrinsic proteins released from thylakoid membranes isolated from both high-CO_2_-grown cells (High) or cells acclimated to low CO_2_ for 2 h (Low). All lanes were loaded with 10 µg protein.

The phosphorylated 30-kDa, protein was immunoprecipitated using affinity-purified antibodies against the Cah3 polypeptide (Figure 4A). In addition, aminoacid sequence analysis shows that the N-terminal sequence of this phosphoprotein coincided with that of the Cah3 polypeptide. Furthermore, this phosphorylated protein was absent in thylakoid membranes from the *cia3* mutant, which lacks the Cah3 polypeptide (*data not shown*).

To obtain evidence that the cross-reaction of the Cah3 polypeptide with the Thr(P) antibodies was due to a true phosphorylation of this protein rather than to a possible unspecific cross-reactivity, we studied the reversion of the phosphorylation signal following treatment with alkaline phosphatase (AP). Figure 4B shows the results of such experiments. The AP treatment of the fraction containing the extrinsic thylakoid polypeptides was fully effective in dephosphorylating the 30-kDa, phosphoprotein (Figure 4B). All these observations clearly indicate that Cah3 is a major extrinsic phosphoprotein of the thylakoid membranes of *Chlamydomonas* that is phosphorylated upon acclimation to low CO_2_ conditions.

### Cah3 is concentrated in the intrapyrenoid thylakoids upon phosphorylation

**Figure 5:**
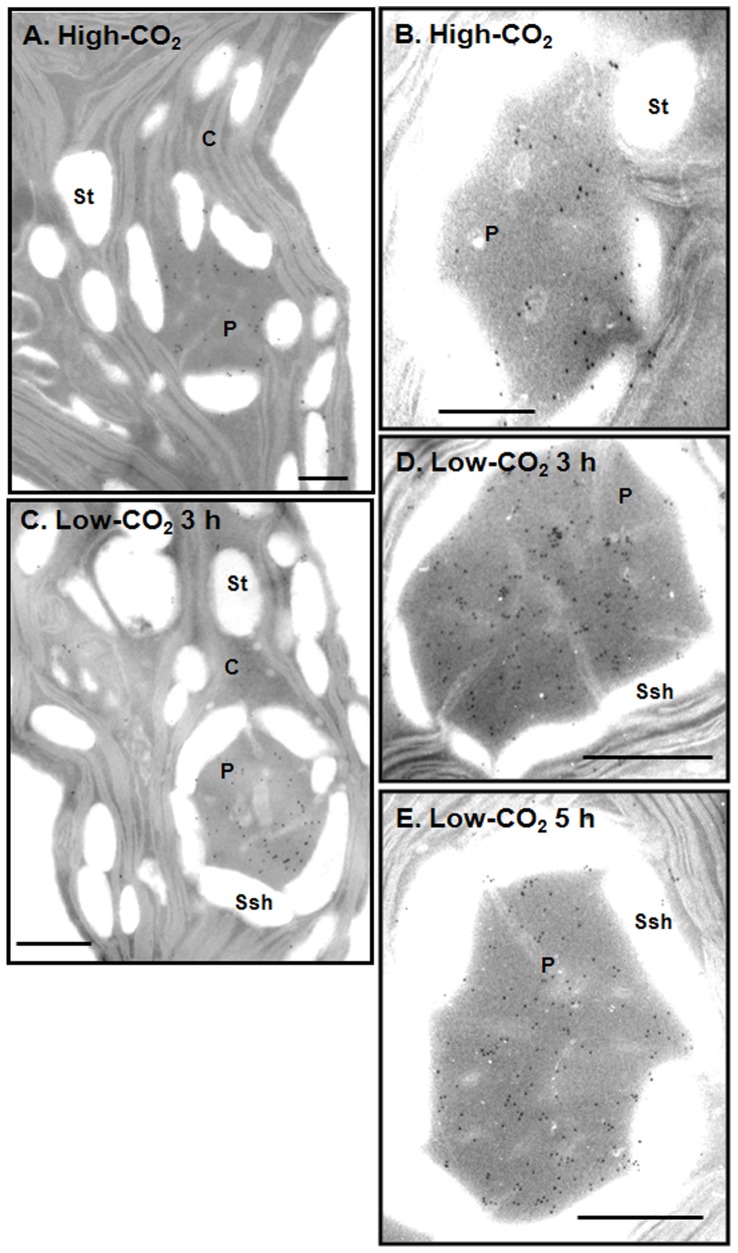
Immunogold labelling of *C. reinhardtii* cells grown on high-CO_2_ or acclimated to low CO_2_ conditions for 2 and 5 h. (**A**) and (**B**) High-CO_2_-grown cells probed with affinity-purified antibodies against Cah3. (**C**) and (**D**) Cells acclimated to low CO_2_ conditions for 3 h and (**E**) pyrenoid of a cell acclimated to low CO_2_ conditions for 5 h, probed with affinity-purified antibodies against Cah3. Bars indicated 0.5 µm. C, chloroplast; P, pyrenoid; Ssh, starch sheath; and St, stroma chloroplast.

It is well established that phosphorylation of the antenna complex components of PSII is affecting their distribution in the thylakoid membranes [25]. To elucidate if phosphorylation of Cah3 is affecting its location in the cell, we carried out immunogold labeling experiments using affinity-purified antibodies against the Cah3 polypeptide. As shown in Figure 5, immunogold labeling densities in the pyrenoid and in the stroma thylakoids depended on the growth conditions. In high-CO_2_-grown cells, immunogold particles were localized to the intra-pyrenoid and stroma thylakoids (Fig. 5A and B). Transferring cells to low CO_2_ conditions resulted in a redistribution of the labeling (Fig. 5C–E). The density of immunogold particles in the stroma thylakoids drastically decreased within the first 3 h of acclimation to low CO_2_ with a concomitant increase in the immunogold density in the thylakoids crossing the pyrenoid (Fig. 5C and D). The same distribution of the immunogold particles, with higher density in the pyrenoid thylakoids, was also observed in cells acclimated to low CO_2_ for 5 h (Fig. 5E). Mitra *et al*. [26] already reported immunolocalization experiments showing that Cah3 is located on the lumenal side of thylakoid membranes including those that penetrate the pyrenoid and showed that immunogold density was more than two times higher in the pyrenoid thylakoids when compared to stromal thylakoids .

When the relative volumes of the pyrenoid and the stroma are taken into account [27], it is clear that the fraction of Cah3 in the pyrenoid region significantly increased in cells acclimated to low CO_2_ for 3 h as compared with high-CO_2_ cells (Table 1). In the latter, only 19% of the total Cah3 was associated with the pyrenoid region, while in low-CO_2_-grown cells 37% of the Cah3 protein was associated with the pyrenoid (Table 1). Similar calculations using data from high-CO_2_-grown cells show that a significant fraction (more than 80%) of the Cah3 is localized to the non-pyrenoid thylakoids in those cells (Table 1). These data imply that the localization of Cah3 in *C. reinhardtii* depends in part on the growth conditions of the organism. In addition, our results indicate that the redistribution of the protein to the pyrenoid occurred simultaneously with the phosphorylation of the Cah3 polypeptide.

**Table 1:** Calculation of Cah3 fraction in both the pyrenoid and the stroma of the chloroplast of both high-CO_2_ cells and cells acclimating to low CO_2_ conditions for 3 and 5 h.

Growth Conditions	Immunogold Density (Gold particles/µm^2^)		Cah3 in Pyrenoid (%)
	Pyrenoid	Stroma	
High CO_2_	31,0±6,54	9,0±2,2	18,8
Low CO_2_ (3 h)	52,3±14,49	5,9±2,0	37,3
Low CO_2_ (5 h)	59,9±16,82	7,0±2,5	36,7

Immunogold labelling experiments were carried out using antibodies against Cah3. Evaluation of labelling was made using the computer program UTHSCSA Image Tool version 3.0. The gold particles associated with the pyrenoid and the stroma were counted and the density calculated on the basis of area. To calculate the fraction of Cah3 in the pyrenoid, the particle density of the pyrenoid or stroma was multiplied by the average volume of the compartment (which is 2.4 µm^3^ and 35.6 µm^3^, respectively, according to [27], giving the total particles for each compartment. The total number of particles in the pyrenoid was divided by the combined number of particles in the pyrenoid and in the stroma. The data shown are the averages ± SD of 30 samples. Preimmune sera gave immunogold densities of less than 2 particles/µm^2^.

**Figure 6:**
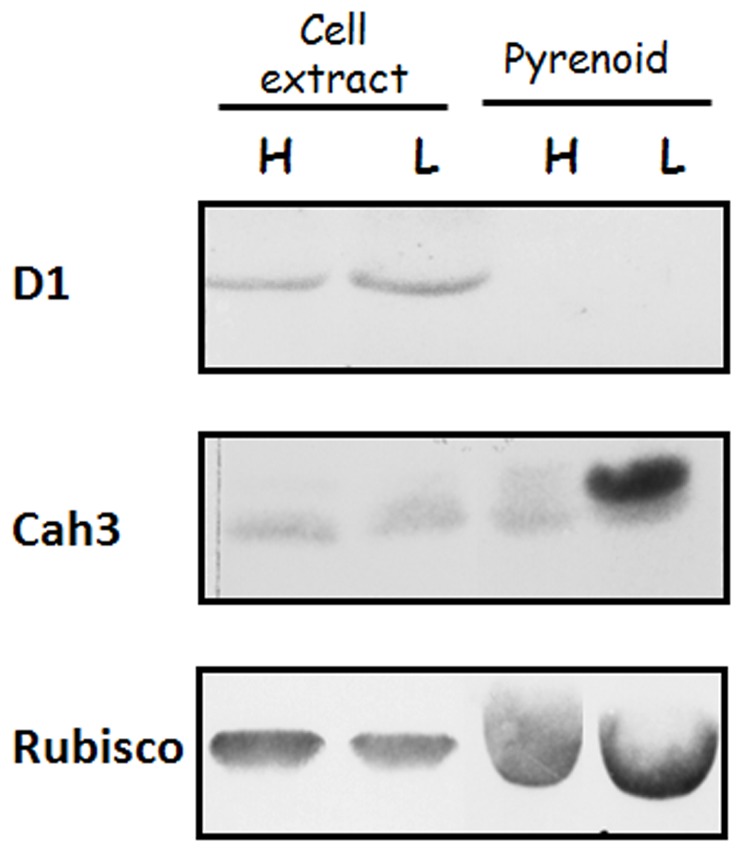
Immunoblot analysis of total cell extracts and isolated pyrenoid fractions from high- (H) and low-CO_2_-grown (L) *Chlamydomonas* cells probed with antibodies against D1 protein of PSII, Cah3 and Rubisco large subunit. All lanes were loaded with 10 µg protein.

It has been recently shown using confocal microscopy of thylakoid auto-fluorescence, that intrapyrenoid thylakoids lack putative PSII fluorescence [28]. Immunoblot analysis of isolated pyrenoid fractions probed with antibodies against the D1 protein of PSII show that this polypeptide was absent in pyrenoids isolated from both high- and low-CO_2_-grown *C. reinhardtii* cells (Figure 6). However immunoblot analysis against Cah3 antibodies clearly confirms that pyrenoid fractions from low-CO_2_-grown cells were enriched in Cah3 when compared to those from high-CO_2_-grown cells (Figure 6). These results indicate that Cah3, which is associated with PSII in high-CO_2_-grown cells [6], [7], is being concentrated in the intrapyrenoid thylakoids, which do not contain PSII, during the acclimation to low CO_2_ conditions.

### The association of Cah3 with PSII also changes during the acclimation to low CO_2_ conditions

Immunogold labeling experiments show that Cah3 was enriched in the PSII-depleted intrapyrenoid thylakoids in low-CO_2_-grown *C. reinhardtii* cells as compared to high-CO_2_-grown cells (37% *versus* 19%). However, a significant portion of the protein was still associated with stromal thylakoids even under low CO_2_ conditions.

Previously, it has been shown that Cah3 is associated with PSII core complex from high-CO_2_-grown *Chlamydomonas* cells [6]. To test if the Cah3 fraction associated with stromal thylakoid membranes in low-CO_2_ cells was also associated with PSII core complexes, we isolated these complexes from cells growing under low CO_2_ conditions for 4 h. Immunoblot analysis shows that Cah3 was still enriched in PSII core complexes when compared to thylakoids and PSII membrane fragments, as it occurs with D1 protein of PSII core complexes used as control(Figure S1).

The PSII core complexes isolated from low-CO_2_ cells were highly active, though their O_2_ evolution rates were lower than those supported by core complexes isolated from high-CO_2_ cells (Table 2). Table 2 also shows the light-saturated O_2_ evolution rates of BBY preparations (PSII membrane fragments, see Materials and methods) from both high- and low-CO_2_-grown cells. In the latter, the O_2_ evolution rates were lower than in the former. Interestingly, when 1 mM HCO_3_^−^ was added to both BBY preparations and PSII core complexes from low-CO_2_ cells, the light-saturated O_2_ evolution was stimulated by ∼15% (Table 2). No stimulation by HCO_3_^−^ was observed in BBY preparations from high-CO_2_ cells (Table 2), as was previously reported by Villarejo *et al* [6]. The HCO_3_^−^ requirement observed in BBY preparations from low-CO_2_-grown *Chlamydomonas* cells resembled the situation in the mutant *cia3*, though in the latter the stimulation by HCO_3_^−^ was higher [6], [7].

**Table 2:** Light-saturated O_2_ evolution rates in both BBY preparations and PSII core complex isolated from high- and low-CO_2_ *C. reinhardtii* cells.

Treatment	O_2_ evolution rates (µmol O_2_/mg Chl/h)	
	High-CO_2_	Low-CO_2_
BBY preparation, control	217±13	189±3
BBY preparation, +1 mM HCO_3_^−^	218±12	212±6
Core complex, control	866±13	805±10
Core complex, +1 mM HCO_3_^−^	870±9	900±30

The light-saturated O_2_ evolution rates in isolated BBY preparations was measured in a buffer containing 20 mM MES-KOH pH 6.5, 300 mM sucrose, and 35 mM NaCl, in the presence of 1 mM DCBQ and 1 mM K_3_Fe(CN)_6_. The light-saturated rates of O_2_ evolution in PSII core complexes were measured in a buffer containing 25 mM MES-KOH pH 6.5, 0.3 M sucrose, 10 mM NaCl and 50 mM CaCl_2_, in the presence of 1 mM DCBQ and 1 mM K_3_Fe(CN)_6_.

**Figure 7:**
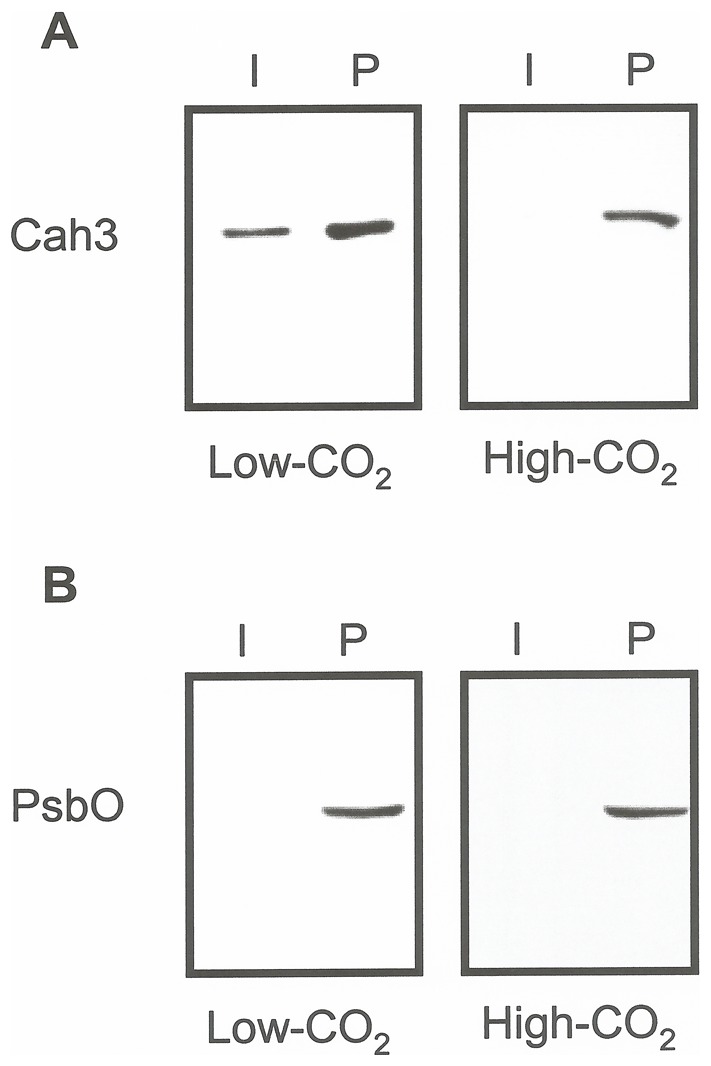
Differential extraction of Cah3 polypeptide from PSII core complexes isolated from *C. reinhardtii* cells grown under high-CO_2_ (A) or acclimated to low CO_2_ conditions for 4 h (B). PSII hydrophobic and hydrophilic proteins were extracted from PSII core complexes using a chloroform/methanol (2∶1, v/v) mixture (see Materials and Methods). Immunoblot analysis of integral (I) and peripheral (P) protein fractions from PSII core complexes were probed with antibodies against Cah3 (Cah3) and PsbO protein (PsbO). The lanes were loaded with 10 µg protein.

The HCO_3_^−^ requirement was not the only difference between PSII preparations from high- and low-CO_2_ *C. reinhardtii* cells. When PSII core complexes from high- and low-CO_2_ cells were subjected to differential extraction of hydrophobic and hydrophylic proteins (Figure 7) we observed that the association of the Cah3 protein with the reaction centre changed depending on the growth conditions. In PSII core complexes from high-CO_2_-grown cells, Cah3 protein was completely extracted in the peripheral fraction (Figure 7A right). However, in PSII fractions from low-CO_2_-grown cells a significant fraction of Cah3 still remained in the integral fraction (Figure 7A left). As a control, we analyzed the distribution of the PsbO polypeptide (Figure 7B). This protein always fractionated in the peripheral fraction, regardless the growth conditions (Figure 7B right and left). These data confirm our previous hypothesis that the association of Cah3 with PSII is changing upon acclimation to low CO_2_ conditions.

It is interesting to note that no cross-reactivity of Thr(P) with any phosphoprotein at 30 kDa was observed in the peripheral fraction of PSII core complexes from both high- and low-CO_2_ cells (*data not shown*). These results indicate that the phosphorylated form of the Cah3 polypeptide was not associated with the PSII core complex.

## Discussion

In this study, we provide strong evidence that Cah3, a lumenal CA associated with the donor side of PSII, is regulated via phosphorylation during the acclimation of *C. reinhardtii* cells to low CO_2_ conditions. Phosphorylation of Cah3 causes partial activation of the enzyme and its redistribution to PSII-depleted thylakoid membranes. Like other thylakoid phosphoproteins, Cah3 seems to be phosphorylated at threonine/serine residues [21], [18]. However, Cah3 differs significantly from all previously characterized thylakoid phosphoproteins and is, to our knowledge, the first characterized lumenal phosphoprotein in any photosynthetic organism, whereas all of the other phosphoproteins are integral membrane polypeptides or confined to the stromal side [18]. The occurrence of phosphorylation events in the thylakoid lumen has been earlier suggested [29], [30] but the relevance of this indication was never elucidated. Our present knowledge of the lumen from a compositional and functional point of view has increased during the last years, mainly due to proteomic analysis approaches using the plant model *Arabidopsis thaliana* (see [31], [32]). Spetea *et al.* [33] have provided clear experimental evidence that the thylakoid lumen contains both nucleotides and enzymes related to nucleotide interconversion processes. And recently Wu *et al.* [34] have reported an O-phospho-L-serine phosphatase activity of AtTLP18.3 in the thylakoid lumen of *Arabidopsis thaliana*. Our finding together with these experimental evidences indicate that the lumen has to be considered as a metabolically active compartment requiring energy and involved in signal transduction mechanisms.

Several potential phosphorylation sites are found in the sequence of the Cah3 protein, including one threonine at position 13 in the N-terminus and two serine residues. In our study only phosphothreonine antibodies cross-react with the Cah3 polypeptide (Figure 3C), indicating that threonine 13 is the phosphorylated residue. According to a structural model for the human α-CA II, the N-terminus of the protein is predicted to be flexible and closed to the active site [35]. In fact, truncations of this domain greatly affect the CA activity [35]. Based on these observations, it can be postulated that phosphorylation of the threonine residue at position 13 in Cah3 may affect activity or binding of the protein to the thylakoid membranes.

CA has long been known to catalyze the reversible hydration of CO_2_, accelerating this reaction by a factor of >10^6^. At present, no evidence is available for any kind of regulation of this well studied enzyme. This work is the first report of a CA being post-translationally regulated via phosphorylation. The question arises about what are the functional implications of this regulatory process. It has been previously shown that Cah3 is associated with the donor side of PSII and is essential for the optimal function of the water oxidizing complex (WOC) when cells are growing under high CO_2_ conditions [6], [7]. However, only after transferring cells to CO_2_ limiting conditions, the Cah3 protein is strongly phosphorylated (Figure 3C). The phosphorylation process correlates with the redistribution of one part of the Cah3 population to PSII-devoid thylakoid membranes (Figures 5 and 6) and this process also correlates with a 5- to 6-fold increase in its enzymatic activity (Figure 1A). A similar increase in thylakoid CA activity has been previously reported in *Chlamydomonas* by using mass-spectrometric and photoaffintiy labeling techniques [19], [20].

As it was mentioned above, acclimation to limiting CO_2_ conditions not only causes phosphorylation of Cah3 but also phosphorylation of other thylakoid proteins. Turkina *et al*, [18] identified and sequenced four phosphopeptides by nanospray-quadrupole-time-of-flight MS from the cells that were transferred to limiting CO_2_. Three phosphorylated peptides belonged to the Lci5 protein, encoded by the low CO_2_ inducible gene 5 (*lci5*) [36], [37]. The other phosphopeptide originated from a protein (UEP) that has not been annotated (this Unknown Expressed Protein is encoded in the genome of *C. reinhardtii*). UEP was found phosphorylated at a serine residue. Multiple phosphorylation of Lci5 may occur at three threonine and four serine residues. Phosphorylation of both proteins occurred strictly at limiting CO_2_ and it required reduction of electron carriers in the thylakoid membrane, but was not induced by light changes (state transitions). Moreover, both proteins were phosphorylated under low-CO_2_ in the *Chlamydomonas stt7* mutant deficient in the light-activated protein kinase Stt7. The latter was suggested as an early adaptive and signaling response of *Chlamydomonas* to the limited environmental inorganic carbon and correlates with the pattern of phosphorylation found for Cah3 in this work.

The photosynthetic apparatus is more and more understood as a flexible molecular machine that can acclimate to metabolic and light fluctuations in a matter of seconds and minutes [38]. Most of these changes seem to be related to phosphorylation processes as can be concluded from the recent work of Lemeille et al. [16]. These authors made the comparison of the thylakoid phosphoproteome of the wild-type strain and the stt7 mutant of Chlamydomonas under state 1 and state 2 conditions. From their analysis it can be concluded that the different thylakoid phosphoproteins could be classified according to the phosphorylation pattern as Stt7-dependent or Stt7-independent, and the latter in turn as dependent or independent of the light conditions, as it was the case for Lci5, which has been shown to be specifically phosphorylated under low-CO2 conditions [18].

The most conspicuous consequence of the phosphorylation of Cah3 is the change in its subcellular location (Fig. 5 and Table 1). The present study demonstrates that the distribution of Cah3 protein along the membranes is variable and depends on the growth conditions to which cells have been acclimated before such an analysis. Our results indicate that the amount of Cah3 in the non-pyrenoid thylakoids drastically decreased within the first 2 h of acclimation to low CO_2_ (Figure 5), when the phosphorylation of Cah3 protein reaches its maximum (Figure 3). Simultaneously, Cah3 is concentrated in the intrapyrenoid thylakoids. About 19% was in the pyrenoid when cells were grown under elevated CO_2_ and about 37% in low CO_2_-grown cells (Table 1) as had been already reported by Mitra et al. [26].

Very recently, Sinetova et al. [39] have confirmed the close relationship between the functional role of Cah3 in the CCM and its localization associated with the pyrenoid thylakoids. These authors related CCM induction and the right distribution of Cah3 associated to pyrenoid thylakoids with an increase in the content of polyunsaturated fatty acids in membrane lipids after low-CO_2_ acclimation. However, in the *cia3* mutant cells, where the immunogold particles against Cah3 appeared evenly distributed throughout the pyrenoid matrix, the increase in the majority of polyunsaturated fatty acids were less pronounced or did not increase at all. The latter could be indicating that the recruitment of Cah3 for lateral movement from the stromal to pyrenoid thylakoids could be more dependent on membrane lipids. In fact we have failed repeatedly in finding the protein partner/s responsible for these lateral movements of Cah3 on the thylakoid membrane.

The fact that the phosphorylated form of Cah3 protein is not associated with PSII complexes leads us to postulate that this is the form that is moving away from PSII and that is concentrated in the intrapyrenoid thylakoids. Pyrenoid morphology undergoes rapid and dramatic changes in response to variations in the CO_2_ concentration in the environment [40]. These changes are correlated with redistribution of Rubisco, which is also more concentrated in the pyrenoid under low CO_2_ conditions [41] as it is the case for Cah3 (Figure 6). Nevertheless, our study clearly shows that the proportion of Cah3 involved in this reorganization (∼20%, Table 1) is, in fact, smaller than that of Rubisco (∼60%, [41]). In *Chlamydomonas*, the bulk of the pyrenoid structure is composed of an electron dense, granular matrix, consisting mainly of Rubisco molecules. Enclosed within this matrix is a system of tubules radiating from a central nexus to the pyrenoid periphery where each tubule exhibits continuity with a stroma thylakoid membrane [42]. This intrapyrenoid system of tubule-like thylakoids, where the phosphorylated form of Cah3 is concentrated, differs from the non-pyrenoid thylakoids and does not contain PSII complexes (Figure 6). Moreover, it has been shown that these intrapyrenoid thylakoids lack putative PSII fluorescence [28], thus supporting the contention that they lack PSII.

It is intriguing why a protein, which is required for the WOC to function optimally under high CO_2_ conditions [6], is partially detached from PSII and concentrated in membranes containing only PSI complexes during the acclimation to limiting CO_2_. Nevertheless, it has been earlier reported, as a consequence of CCM induction, a partial decrease (≈20%) in functional PSII reaction centers, an increase in the activity of PSI *versus* PSII and an increased cyclic electron transport around PSI [43].

The present study provides strong evidence that Cah3 is involved in this reorganization of the photosynthetic light reactions. Our results shows that the partial detachment of Cah3 from PSII is correlated with a partial decrease in the O_2_ evolution rates of PSII core complexes. It is interesting to note that this decrease is of the same order as has been previously reported [43]. In addition, PSII core complexes isolated from low-CO_2_-grown cells resembles those, isolated from the mutant *cia3*, which lacks an active Cah3, and they required bicarbonate for reaching maximum activity [6]. However, the HCO_3_^−^ requirement in PSII preparations from low-CO_2_-grown cells is much smaller than in preparations from the mutant *cia3*, where up to half of the WOCs were non-functional (see [6]). The degree of decrease in functional PSII reaction centers and the HCO_3_^−^ requirement may be correlated with the proportion of Cah3 protein that is detached from PSII.

We postulate that Cah3 is acting as a regulator that allows adjusting the activity of PSII to the amount of the ultimate electron acceptor CO_2_. In high-CO_2_-grown cells, where the high CO_2_ concentration is not limiting photosynthesis, Cah3 is associated with the WOC of PSII and allows the electron donation to be fast enough for an optimal function of PSII and hence reduction of CO_2_ [6]. When cells are acclimating to limiting CO_2_ conditions, at the same light intensity, they will experience an initial stress situation where the low CO_2_ concentration is limiting photosynthesis. This suggests that the photosynthetic apparatus will be exposed to a relatively higher excitation pressure compared with high-CO_2_-grown cells, and this will cause over-reduction of the plastoquinone pool. Under this situation, Cah3 is phosphorylated and partially detached from PSII. The concentration of Cah3 in the intrapyrenoid thylakoids, which are surrounded by active Rubisco molecules, together with its activation will provide locally high CO_2_ concentrations to the carboxylating enzyme. This redistribution of Cah3 and the induction of the CCM will help to overcome the initial CO_2_ limitation and the survival of the cell. This model will fit with that predicted by Raven [10] and will explain the postulated dual role of Cah3 in *C. reinhardtii* cells.

The driving force for the movement of Cah3 between the non-pyrenoid and the intrapyrenoid thylakoids is at present unknown. Another challenge is to understand the observed differences in the binding properties of Cah3 to PSII between high- and low-CO_2_-grown cells (Figure 7). One possible explanation is that changes in the organization of PSII core complex taking place during the acclimation to low CO_2_ conditions could affect the binding properties of the Cah3 polypeptide to PSII, without changing the binding of the other OEC proteins. In fact, it has been reported [44] that the OEC polypeptides, PsbO, PsbP and PsbQ, directly bind to PSII in *C. reinhardtii*. In contrast to the situation in higher plant PSII, the binding of each OEC subunit is independent of the other extrinsic proteins [44]. A more detailed structural analysis of the PSII core complexes from low-CO_2_-grown cells will be required to solve this question.

## Materials and Methods

### Strains and growth conditions

*C. reinhardtii* cell wall-deficient mutant 92 (*cw92*) which is regarded as the standard wild type in photosynthesis studies, was obtained from the *Chlamydomonas* Culture Collection at Duke University, Durham, NC, USA. The cell wall-deficient, high-CO_2_-requiring *cia3* double mutant was kindly provided by J. V. Moroney (Louisiana State University, Baton Rouge, LA) [9]. All strains were grown in batch cultures at 25°C under a continuous irradiance of 150 µmol/m^2^/s supplied from cool, white fluorescent lamps. Cells were cultured in minimal medium [45], under aeration with air enriched with 5% CO_2_ (high-CO_2_-grown cells). For experiments in which cells were shifted from high CO_2_ to low CO_2_ (0.035% CO_2_), cells were previously cultured in high CO_2_ conditions and then shifted to ambient CO_2_ for various times (low-CO_2_-grown cells).

### Isolation of PSII membrane fragments, PSII core complexes, and pyrenoids

Thylakoid membranes were isolated from high- and low-CO_2_-grown Chlamydomonas cells according to [46]. The PSII membrane fragments (BBY preparations, from Berthold, Babcock and Yocum PSII membrane fragments) were isolated as previously described from higher plants [47] PSII core complexes were isolated from BBY preparations of wild-type cells according to [48]. All buffers were supplemented with 10 mM NaF, a phosphatase inhibitor. Pyrenoids were isolated according to the method of [49]. Chlorophyll concentrations of algal subcellular fractions were determined spectroscopically after extraction in absolute methanol [50].

### Differential extraction of extrinsic proteins from thylakoids and PSII core complexes

Differential extraction of Cah3 from thylakoid membranes was performed by salt washing. Thylakoid membranes were resuspended to a chlorophyll density of 1 mg Chl/ml in incubation buffer containing 50 mM Tris-HCl buffer pH 8.0, 1 mM EDTA, and supplemented with 1 M NaCl. Samples were incubated in the washing solution for 1 h at 4°C in darkness with gentle stirring. After incubation, samples were centrifuged. The pellet was washed twice with buffer containing 50 mM Tris-HCl buffer pH 8.0, 400 mM sucrose, and 10 mM NaCl, and then resuspended in the same buffer. The supernatants were desalted by serial dilution with the salt-free buffer by centrifugation in Centriprep YM-10 tubes (Millipore). Each desalted sample was then concentrated 15 times by centrifugation in the same tubes overnight. All buffers were supplemented with 10 mM NaF, a protein phosphatase inhibitor.

PSII hydrophobic and hydrophylic proteins were extracted from PSII core complexes using a chloroform/methanol (2∶1, v/v) mixture [51], [52]. Briefly, PSII core complex sample was slowly diluted in 6 ml of cold chloroform/methanol (2∶1, v/v) solution. The resulting mixture was stored for 15 min on ice before centrifugation. The proteins insoluble in this phase (peripheral proteins) were recovered as a white pellet and resuspended in 2× SDS-PAGE sample buffer. The organic phase, that contained integral proteins was precipitated with ice-cold acetone and resuspended in 2× SDS-PAGE sample buffer. Both fractions were analyzed by SDS-PAGE and western blot. For *in vivo* analysis of the phosphorylation pattern of extrinsic proteins, thylakoid membranes were incubated in medium containing 50 mM Tris-HCl pH 7.5, 150 mM NaCl, 1 mM MgCl_2_, and 0.05% (v/v) Triton X-100. After 20 min incubation on ice, the membranes were pelleted and the supernatants were analyzed by SDS-PAGE and western blots.

### *In vitro* dephosphorylation of Cah3

The fraction obtained after treatment of thylakoid membranes with incubation medium containing 0.05% Triton X-100 (100 to 150 µg protein) was incubated for 10 min at 30°C. After this incubation, the sample was divided into two halves. One was treated by adding 30 units of alkaline phosphatase (type VII-T, Sigma) for 15 min at 30°C; the other was used as a control. The reaction was terminated by adding an equal volume of 2× SDS-PAGE sample buffer. The completion of the dephosphorylation reaction was checked by analyzing samples by western blot.

### Immunoprecipitation experiments

Samples obtained after washing thylakoid membranes from low-CO_2_ cells were diluted to 300 µl by using ice-cold immunoprecipitation buffer containing 50 mM Tris-HCl pH 7.5, 150 mM NaCl, 2% (v/v) Igepal CA-630, and a protease inhibitor cocktail (Complete, Roche Diagnostics, Mannheim, Germany). 10 µl of affinity-purified anti-Cah3 antibodies were subsequently added, and samples were mixed by gentle shaking for 2 h at room temperature. 20 µl 10% (v/v) protein A-Sepharose CL-4-B beads (Sigma) were added, and samples were mixed for 4 h at room temperature. Protein A beads were collected by centrifugation and washed twice with 1 ml washing buffer containing 50 mM Tris-HCl pH 7.5, 150 mM NaCl, 0.5% (v/v) Igepal CA-630, 0.1% (w/v) SDS. The beads were boiled for 5 min in 2× SDS-PAGE sample buffer and samples analyzed by western blot.

### Carbonic anhydrase activity measurements

The activity of CA was potentiometrically determined by measuring the time for the pH to decrease from 8.2 to 7.4, at 2°C, in a sample of 2 ml of 75 mM phosphate buffer (pH 8.2), containing 1 mM EDTA and 0.1 mM dithiothreitol (DTT), upon addition of 2 ml of ice-cold CO_2_-saturated distilled water [53]. One Wilbur-Anderson unit (WAU) [54] was defined as: WAU = (t_0_/t_c_-1) 10, where t_0_ was the time for the pH change with buffer controls and t_c_ was the time obtained when CA-containing samples were added.

### Photosynthetic response curves

Photosynthetic O_2_ evolution was measured at saturating white light and 25°C using a Clark-type oxygen electrode (CB1D, Hansatech, Norfolk, UK). Cells were resuspended in 20 mM CO_2_-free buffer HEPES-KOH buffer (pH 7.2) at a chlorophyll density of 2–5 µg Chl/ml. The cell suspensions were then placed in the illuminated electrode chamber and allowed to consume the dissolved Ci of the medium and the intracellular Ci pool until no net photosynthesis was observed. HCO_3_^−^ at the indicated concentrations was added and the rate of O_2_ evolution measured.

### *In vitro* measurements of electron flow

The light-saturated electron flow through PSII in BBY preparations was measured in a buffer containing 20 mM MES-KOH pH 6.5, 400 mM sucrose, and 35 mM NaCl, in the presence of 1 mM K_3_Fe(CN)_6_, and 1 mM DCBQ. The light-saturated rates of oxygen evolution in PSII core complexes were measured in a buffer containing 25 mM MES-KOH (pH 6.5), 300 mM sucrose, 10 mM NaCl, and 50 mM CaCl_2_, in the presence of 1 mM DCBQ and 1 mM K_3_Fe(CN)_6_ as described by [55].

### Western blot analysis

Isolated thylakoid membranes, PSII membrane fragments, total cell proteins, and PSII core complexes were separated on 12% SDS polyacrylamide gels [56]. After electrophoresis, proteins were blotted onto a nitrocellulose membrane. Immunoblotting was performed as described in the protocol supplied by BioRad Laboratories. Horseradish peroxidase-labeled secondary antibodies and enhanced chemiluminescence (ECL, Amersham International) were used to detect the antibody-antigen conjugate.

### Electron microscopy and immunolocalization

For immunolabeling experiments, cells were fixed with 3% freshly depolymerized paraformaldehyde and 0.3% glutaraldehyde in PBS for 1 h, transferred to 3% paraformaldehyde and stored at 4°C over night. The pellets were infused with 20% polyvinylpyrrolidone in 2.3 M sucrose in PBS for 3 h, mounted on stubs and subsequently frozen in liquid nitrogen. Ultrathin sections were cut and mounted on formvar coated niquel grids.

Preincubation of sections was performed in a solution of 0.01% blocking reagent (Boehringer Mannheim) and 0.75% glycine for 15 min. The sections were incubated for 1 h with the primary antibodies (1∶200 dilution) or with preimmune serum diluted similarly as a control, washed for 30 min in PBS/blocking reagent, and incubated with goat anti-rabbit gold conjugate (Biocell) (diluted 1∶50) for 1 h. Finally, the sections were washed in PBS and distilled water and embedded in 2% methyl-cellulose and 0.3% uranyl acetate.

Evaluation of immunogold labeling was made using the computer program UTHSCSA Image Tool version 3.0 (Department of Dental Diagnostic Science, University of Texas Health Science Center, San Antonio, Texas). The gold particles associated with the pyrenoid were counted, as were the particles in the same size area in the cytosol and stroma regions of the individual cells.

### Estimation of relative transcript levels with RT-PCR

To determine specifically the relative transcript levels of the *cah3* gene, RT-PCR assays were performed. Total RNA was isolated from 2 ml culture by using the Trizol™ reagent according to the manufacturers protocol (Life technologies, US). 2,5 ng total RNA from wild-type and mutant *cia3* were preheated to 70°C for 20 minutes prior to the RT-PCR reactions. Advantage One-step RT-PCR Kit (Clontech Laboratories, Inc CA, USA) was used and 10 µl of each reaction mix were run on a 1% agarose gel. The following primers were used for the different transcripts: *tubB2*, 5′–CTTGTTCTGCACGTTCAGC–3′ and 5′–AAGCAGATGTCGTACAGG–3′; *cah3*, 5′-GTTCATTGGCAACATGGAGC-3′ and 5′-TGGCGTATGTCTTGTTGTCG-3′.

## Supporting Information

Figure S1 **Association of Cah3 polypeptide with PSII core complexes from high- and low-CO_2_-grown** ***C. reinhardtii*** **cells.** Immunoblot analysis of thylakoid membranes (Thy), BBY preparations (BBY), and PSII core complexes (Core) from cells of *C. reinhardtii* with antibodies raised against the over-expressed Cah3 polypeptide (Cah3) and D1 protein of PSII. The lanes were loaded with 10 µg protein.
(TIF)
