# Supplementary figures and images for: Phosphorylation Controls the Localization and Activation of the Lumenal Carbonic Anhydrase in Chlamydomonas reinhardtii
Source: PLoS One. 2012 Nov 6;7(11):e49063. doi: 10.1371/journal.pone.0049063 (PMC3490910; doi:10.1371/journal.pone.0049063)

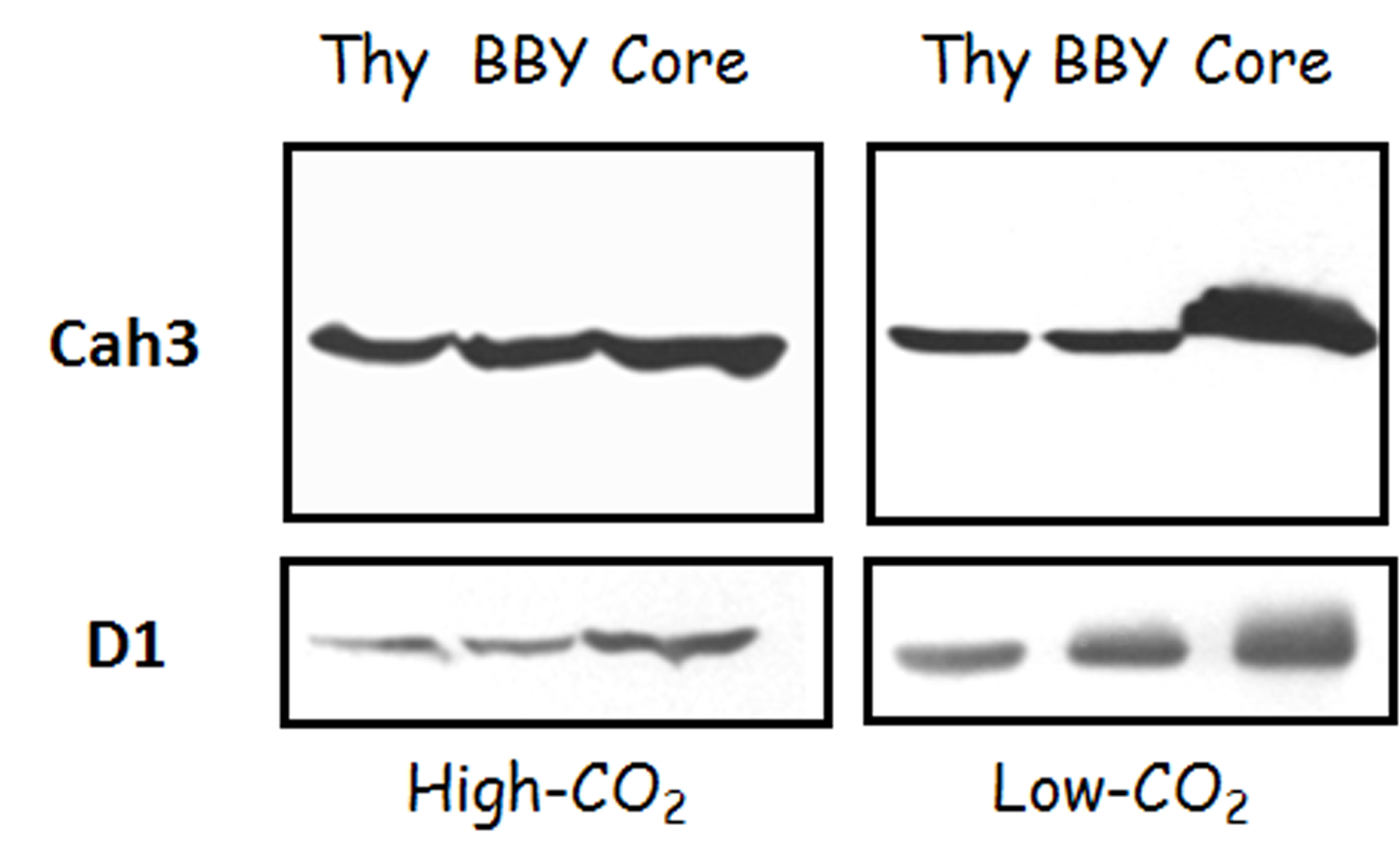

Supplement: Figure S1 — Association of Cah3 polypeptide with PSII core complexes from high- and low-CO2-grown C. reinhardtii cells. Immunoblot analysis of thylakoid membranes (Thy), BBY preparations (BBY), and PSII core complexes (Core) from cells of C. reinhardtii with antibodies raised against the over-expressed Cah3 polypeptide (Cah3) and D1 protein of PSII. The lanes were loaded with 10 µg protein. (TIF) [file pone.0049063.s001.tif]
